# Supplementary material for: Trajectories of self-kindness, common humanity, and mindfulness during the COVID-19 pandemic: A person-oriented multi-trajectory approach
Source: PLoS One. 2023 Dec 18;18(12):e0292522. doi: 10.1371/journal.pone.0292522 (PMC10727429; doi:10.1371/journal.pone.0292522)
Supplement: Attachment — Submitted filename: Response to reviewers.pdf [file pone.0292522.s005.pdf]

Reviewers' comments:

Reviewer's Responses to Questions

Comments to the Author

1. If the authors have adequately addressed your comments raised in a previous round of review and you feel that this manuscript is now acceptable for publication, you may indicate that here to bypass the “Comments to the Author” section, enter your conflict of interest statement in the “Confidential to Editor” section, and submit your "Accept" recommendation.

Reviewer #3: All comments have been addressed

Reviewer #4: (No Response)

Reviewer #5: (No Response)

Reviewer #6: (No Response)

Reviewer #7: (No Response)

2. Is the manuscript technically sound, and do the data support the conclusions?

The manuscript must describe a technically sound piece of scientific research with data that supports the conclusions. Experiments must have been conducted rigorously, with appropriate controls, replication, and sample sizes. The conclusions must be drawn appropriately based on the data presented.

Reviewer #3: Yes

Reviewer #4: Partly

Reviewer #5: Partly

Reviewer #6: (No Response)

Reviewer #7: Partly

3. Has the statistical analysis been performed appropriately and rigorously?

Reviewer #3: Yes

Reviewer #4: I Don't Know

Reviewer #5: I Don't Know

Reviewer #6: (No Response)

Reviewer #7: Yes

4. Have the authors made all data underlying the findings in their manuscript fully available?

The PLOS Data policy requires authors to make all data underlying the findings described in their manuscript fully available without restriction, with rare exception (please refer to the Data Availability Statement in the manuscript PDF file). The data should be provided as part of the manuscript or its supporting information, or deposited to a public repository. For example, in addition to summary statistics, the data points behind means, medians and variance measures should be available. If there are restrictions on publicly sharing data—e.g. participant privacy or use of data from a third party—those must be specified.

Reviewer #3: Yes

Reviewer #4: Yes

Reviewer #5: Yes

Reviewer #6: (No Response)

Reviewer #7: Yes

5. Is the manuscript presented in an intelligible fashion and written in standard English?

PLOS ONE does not copyedit accepted manuscripts, so the language in submitted articles must be clear, correct, and unambiguous. Any typographical or grammatical errors should be corrected at revision, so please note any specific errors here.

Reviewer #3: Yes

Reviewer #4: Yes

Reviewer #5: Yes

Reviewer #6: (No Response)

Reviewer #7: Yes

6. Review Comments to the Author

Reviewer #3: The authors have thoroughly addressed all the edits requested by previous reviewers. The paper is methodologically sound and provides an interesting addition to the literature. I suggest this paper is accepted with no further revisions.

Response: We would like to thank reviewer #3 for his/her positive and encouraging feedback on our revised manuscript.

Reviewer #4: Using a longitudinal research design, the authors aim to identify and describe the individual trajectories of self-kindness, common humanity, and mindfulness during the first eight months of the COVID-19 pandemic. The paper is interesting in its objective, but I have some concerns about the method chosen to evaluate the constructs and the general organization of the paper. In addition, some comments will be mainly directed at improving the organization of the paper, which appears difficult to follow and, in some pieces, redundant and not flowing.

Response: We would like to thank reviewer #4 for his/her constructive and valuable comments on our work.

The introduction section appears complicated to follow, with continuous references to the study's objectives and hypotheses that should be clarified once with the support of the scientific literature.

Response: Based on the reviewer's comments, we have improved and streamlined the introduction. For instance, we have included newer references, rewritten the mental health section, renamed some subheadings, and provided additional modifications. Below, we detailed all of these changes. We hope that this newly improved version is clearer for you and for future readers.

The references to mental health during the covid era are referred mainly to 2020, and some more recent papers should be presented, as much literature has been produced.

Response: As suggested, we have included more recent references and several meta-analyses in the mental health section. Specifically, we wrote (page 3): "The COVID-19 pandemic and its consequences have negatively affected mental health worldwide. In a cross-national study involving 48 countries, Gamonal-Limcaoco et al. (2022) found high levels of negative emotions in the general population, with higher levels among women, young adults and those who expressed concern about getting infected. Consistent with these findings, several other studies have documented the negative impact of COVID-19 on emotional experiences. Based on the pooled effect sizes of 19 studies, Ernst et al. (2022) found an overall small increase in loneliness since the start of the pandemic (see also Buecker & Horstmann, 2021 for similar results). Similarly, in their systematic review and meta-analysis, Salari et al. (2020) highlighted the prevalence of stress, anxiety, and depression among the general population during the COVID-19 pandemic. In the same vein, two meta-analyses demonstrated that the COVID-19 pandemic increased negative emotions and disrupted sleep quality (Jahrami et al., 2021; Oliveira Carvalho et al., 2021). Moreover, Satici et al. (2020) found that the fear of COVID-19 was positively associated with psychological distress and negatively associated with life satisfaction. Other studies have demonstrated an increase in psychological and mental health disorders globally during the COVID-19 pandemic (Leung et al., 2022)."

The authors should reorganize the introductory section by clarifying the constructs investigated and the relationship between them and then subsequently define objectives and hypotheses in a clear and defined way and consequently define the identified method. I suggest integrating the paragraph Compassionate Self-Responding and Sociodemographic Factors. The reading and fluency of the paper can benefit from this change.

Response: Thank you for this suggestion. We have integrated the "Compassionate Self-Responding and Sociodemographic Factors" in the introduction to facilitate the reading. We believe this change indeed enhances fluency.

Regarding the method section, I am uncertain about the authors' choice of using Reynolds' adaptation for emotion analysis. Could the authors clarify the choice regarding the use of this tool? The authors talk about mental health, but they assessed the degree experienced in certain emotions, which does not measure mental health. I suggest the concept of well-being instead.

Response: Thank you for bringing this error to our attention. We indeed relied on the items used by Reynolds and colleagues (2007). However, we cited the wrong paper in the reference section, which may have caused confusion. We have now cited the appropriate reference (see below). In this context, we measured emotional well-being, assessed via positive and negative emotions, as a common indicator of mental health.

Reynolds, D. L., Garay, J. R., Deamond, S. L., Moran, M. K., Gold, W., & Styra, R. (2007). Understanding, compliance and psychological impact of the SARS quarantine experience. *Epidemiology & Infection, 136*(7), 997-1007. https://doi.org/10.1017/S0950268807009156

Define whether "gender" refers to "sex assigned at birth" or "gender identity."

Response: In the measures section, we now write: "In the first section of Wave 1, the participants provided the main sociodemographic information. Specifically, they were asked to indicate their age, gender identity, province of residence, profession, education, and to answer COVID-19 related questions."

My primary concern is about the validity and reliability of the Compassionate Self-Responding measure because authors refer to "dimensions" but have only measured three elements (items). this aspect needs to be stressed more.

Response: We thank you for this comment and the opportunity to clarify and enhance our analysis. First, we now clarify that each dimension of self-responding was assessed with a single item in the "Introduction" and "Method" sections. Additionally, we are now referring to "facet" instead of "dimension" throughout the manuscript. Most importantly, we have collected additional data to test for their convergent validity. Specifically, we wrote (page 8): "To assess the convergent validity of the three positive facets of self-compassion, we conducted an additional data collection (Wave 11, $N = 1671$). This data collection included the three adapted single items tailored to the COVID-19 context, as well as their respective single items from the original scale. The correlation results presented in Table 2 demonstrated significant and strong associations between the COVID-19 items and the original items, supporting the convergent validity of our scale ($r_{\text{self-kindness}} = .56, p < .001, r_{\text{common humanity}} = .58, p < .001, r_{\text{mindfulness}} = .53, p < .001$)."

I suggest summarizing the part explaining the analyses because it is verbose.

Response: We agree with you that the "Statistical Modeling" section is complex. Indeed, during the first round of revision, some reviewers requested more detailed information about the analysis strategy which led us to expand this part, in order to provide as much information as possible for scholars who wish to replicate our findings using similar methods. However, we agree with your suggestion and have trimmed (a little bit) parts when possible.

The discussions are centered on the results obtained however need to fully clarify the limitation found in the method.

Response: Thank you for this important comment. We have created a limitations section in which we carefully detailed several limitations including (a) operationalization of the scale, (b) assessment of mental health, and (c) correlational design. We believe that these points address the main limitations of the present work. If you believe that other elements should be included, we will be happy to do so.

Thank you again for your careful revision.

Reviewer #5: This paper from Canada explores trajectories of compassionate self-responding (CSR) during the COVID19 pandemic using 10 waves of data collection in a longitudinal research design. In addition it aims to compare this data with socio demographic variables and mental health indicators. The study team have done an excellent work producing an important study finding 4 clusters of individual trajectories of CSR. I feel this is an important addition into the body of literature in positive psychology especially in the context of the COVID19 pandemic. The claims made in the paper with respect to the different trajectories and their correlation with gender and age seem sound and deserves publications. However, I feel the paper will benefit by addressing the following comments prior to publication.

Response: We would like to thank reviewer #5 for his/her very detailed and useful revision of the manuscript.

1. One of the 3 purposes of the research is to look at correlation between the trajectories of CSR and mental health indicators. This is linked to Hypothesis 3 "We hypothesize that high multi-trajectory groups will be positively associated with indicators of good mental health (negative emotions, happiness, sleep quality and quantity, and life satisfaction)" However, despite it being a longitudinal study they have collected indicators associated with mental health only at the end of the study, namely, participants' negative affect, happiness, life satisfaction, sleep quantity and quality. Further, some variables such as sleep quality and quantity capture only that of the last 24 hours which is likely to give limited information. It is unclear why mental health variables were collected only in the last wave of the study despite the longitudinal design. Hence, I feel the purpose and hypothesis 3 and the associated conclusions cannot be fairly made and the phrasing of associated claims is not fully supported by the data and analyses. Although this is explicitly stated as a limitation, unless there is further evidence, I feel the claims under purpose and hypothesis 3 may be modified.

Response: We agree that this is a good point to consider, and in designing the study we opted for the approach we presented in our paper. In the present study, trajectories were intentionally designed to span time 2 to time 9, encompassing predictors from time 1 and evaluating outcome variables at time 10, two months post-survey. This strategic approach aimed to establish preliminary evidence for causal inferences while mitigating overlaps (Nagin et al., 2018). It provides answers to a specific research question "Do self-compassion trajectories predict mental health indicators?" As you pointed out, another research question that would be important to examine is whether trajectories of self-compassion vary jointly with trajectories of mental health indicators. Future studies could be designed to explore joint trajectories or incorporate time-varying covariates to gain a comprehensive perspective on temporal dynamics between compassionate self-responding trajectories and mental health indicators. We have made this change in the discussion (see pp. 16-17).

2. The authors describe CSR as falling under the global construct of self-compassion and having 3 interconnected dimensions –positive and negative. The authors also quote Neff, 2003b, saying the original Self-Compassion Scale has 26 items. The study uses only 3 positive dimensions and of the 26 items authors choose only 3 items. The justification for this is in the Limitations as "This was based on the highest factor loadings from the original validation study which we then adapted to the context of the pandemic". This justification needs to be moved to earlier and expanded further. It is also unclear whether this new scale was validated.

Response: We have clarified this in the introduction (see pages 3 and 4). Most importantly, we have collected additional data to test for their convergent validity. Specifically, we wrote: "To assess the convergent validity of the three positive facets of self-compassion, we conducted an additional data collection (Wave 11, *N* = 1671). This data collection included the three adapted single items tailored to the COVID-19 context, as well as their respective single items from the original scale. The correlation results presented in Table 2 demonstrated significant and strong associations between the COVID-19 items and the original items, supporting the convergent validity of our scale ($r_{\text{self-kindness}} = .56, p < .001, r_{\text{common humanity}} = .58, p < .001, r_{\text{mindfulness}} = .53, p < .001$)."

Table 2. Means and correlation matrix of all variables measured at Wave 11 (*N* = 1671)

	Mean (SD)	1	2	3	4	5	6
1. Self-Kindness COVID-19	6.91 (2.03)	-	0.69*	0.67*	0.56*	0.50*	0.48*
2. Common Humanity COVID-19	6.76 (2.15)	-	-	0.60*	0.48*	0.58*	0.38*
3. Mindfulness COVID-19	7.22 (1.95)	-	-	-	0.51*	0.41*	0.53*
4. Self-Kindness	6.91 (1.95)	-	-	-	-	0.59*	0.62*
5. Common Humanity	6.71 (2.19)	-	-	-	-	-	0.49*
6. Mindfulness	7.33 (1.92)	-	-	-	-	-	-

***Note*: * Correlation is significant at the .001 level.**

We are also attaching a Word version of this letter to make it easier to read this new table.

3. It is unclear why they omitted all negative items. This is despite the authors stating that "However, these studies focused on the combined effects of compassionate self-responding and its negative counterparts (i.e., self-judgment, isolation, and over-identification) such that it is not clear if these associations were due to the

positive or rather the negative dimensions of self-compassion”. Based on this argument it would have made sense to add some of the negative items as well. This needs to be explained.

Response: We agree that including the negative aspects of self-compassion would have been interesting. We had decided to focus on positive aspects of self-compassion during the COVID-19 pandemic because positive aspects that foster kindness, acceptance, and affection toward the self tend to play a major role in self-compassion interventions (Egan et al., 2022). This justification is presented on page 4. We also clarified our argument on pages 3-4 to focus solely on compassionate self-responding.

4. The study claims to have looked at ‘context’. In addition there is multiple mentions of ‘COVID19 events’ (e.g.: Intro says “Accordingly, we focused on compassionate self- responding while facing COVID-19 events”.) However, it’s unclear why they have not included any variables related to COVID-19 events in the survey (such as COVID19 infection of self, COVID19 infections of loved ones and news of death). The claims regarding the correlations seem to be based solely on Figure 3 where they have looked at 'daily new confirmed COVID-19 cases per million people in Canada'. Hence, I feel claims related to any association between the self-compassionate responding and COVID19 related events need to be modified to clarify that the correlations between COVID19 and self-compassionate responding is based on the timing of data collection, national level daily new confirmed COVID-19 cases and not specific individual COVID19 related events.

Response: As suggested, we have modified the word “events” when necessary and improved our analysis by including COVID-19 related variables. In this context, we used a new analysis strategy and reported it within the manuscript. Specifically:

“COVID-19 related variables. Participants were asked to answer two items assessing fear of infection (“How concerned are you about getting very sick with the virus”) and fear for others (“How concerned are you about a loved one or friend getting very sick with the virus”). Fear of infection and fear for others were positively correlated ($r = .61, p < .001$) at Wave 1.” Note that we initially included infection status but given that only 5 persons got infected in the whole sample, we decided to omit this variable from the analysis.

“Associations between Trajectory Groups, Sociodemographic Factors, and COVID-19 Related Variables. In step 2, we used the MULTRISK function of PROC TRAJ from SAS software to analyze sociodemographic risk factors (i.e., age and gender) and COVID-19 related variables (e.g., fear of being infected) to identify those that could differentiate compassionate self-responding multi-trajectory groups (Hypothesis 2).”

“Finally, individuals who feared infection were more likely to belong to the highest compassionate self-responding trajectory groups. Interestingly, when the fear of infection extended to the family and friends, no difference was observed between trajectory groups.”

Table 4. Factors associated with trajectory groups (Step 2)

	Parameter	Estimate	Standard error	T for H0	Prob > |T|
Low (Reference category)	-	-	-	-	-
Low vs. Moderate Low	Constant	1.05390	0.85743	1.229	0.2190
	Age	0.15162	0.20514	0.739	0.4599
	Gender	-0.01356	0.55404	-0.024	0.9805
	**Fear of infection**	**0.32009**	**0.09584**	**3.340**	**0.0008**
	Fear for family	-0.10399	0.10075	-1.032	0.3020
Low vs. Moderate High	Constant	-0.69584	0.96371	-0.722	0.4703
	**Age**	**0.44590**	**0.19604**	**2.275**	**0.0230**
	Gender	-0.31228	0.52878	-0.591	0.5548
	**Fear of infection**	**0.26591**	**0.10191**	**2.609**	**0.0091**
	Fear for family	0.04458	0.10876	0.410	0.6819
Low vs. High	**Constant**	**-4.15005**	**1.44934**	**-2.863**	**0.0042**
	**Age**	**0.48301**	**0.22609**	**2.136**	**0.0327**
	Gender	-0.43107	0.58894	-0.732	0.4642
	**Fear of infection**	**0.32323**	**0.11107**	**2.910**	**0.0036**
	Fear for family	0.21401	0.16973	1.261	0.2074

In the discussion, we wrote this: "To go further, our study took a closer look at the impact of fear of infection on compassionate self-responding trajectories. Interestingly, we observed a distinction between personal fear of infection and fear extended to family members and friends. While personal fear was linked to higher compassionate self-responding, the extension of fear to others seemed to negate this effect. This suggests that facing the direct threat of COVID-19 prompted individuals to adopt a more self-compassionate stance, possibly as a coping mechanism in the face of adversity. The lack of significant association between fear extended to family members and compassionate self-responding trajectories suggests that while personal fear may lead to greater self-compassion, fear for other people may generate broader compassion rather than compassion for oneself."

Ultimately, we have modified Figure 1 accordingly.

Overall this is an important study that brings useful insights about trajectories of SCR. Hence, I hope these comments are useful to refine the paper so that the important findings about the clusters of individual trajectories of SCR and their correlation with age and gender are shared in a fair manner. Thank you for this opportunity.

Response: Thank you very much for your encouraging words and valuable input about our work. We believe our work has greatly improved and we are looking forward to reading your new feedback on this revised version.

Reviewer #6: (No Response)

Reviewer #7: Thank you for the opportunity to review this paper. I think it is an important area of research in order to discover positive key factors that help people better cope with the psychological challenges associated with the COVID-19 pandemic. However, there are a number of edits that I recommend be made before a possible publication. They are highlighted below in the order in which they appear.

Response: We thank reviewer #7 for his/her pertinent and helpful comments on the manuscript.

- Verbal tenses should be revised all over the manuscript and conjugate in the past. In introduction authors wrote "in this challenging time" or "current pandemic", I suggest to refer to the past;

Response: Thank you for this useful comment. The manuscript was edited for English language usage and grammar by a native English-speaking proofreader who reviewed the entire manuscript. We hope the result will meet your expectations.

- I suggest to move the aims reported in the first section of introduction ("The purpose … social change.") to the end and under the subheading "Overview and hypothesis", summarize the section e aims and changing the subheading title in – i.e. – "Aims and hypothesis"; moreover, there is a part that is a result: "in a large representative sample of Canadians (N = 2458 from an initial sample size of 3617 participants). "

Response: Thank you for this thoughtful suggestion. We have renamed the section "Overview of the Present Study and Hypotheses" with "Aims and Hypotheses". After careful consideration, we decided to keep a short summary at the beginning of the introduction but removed the part you suggested from the paragraph.

- In introduction, talking about gender, authors write "This may explain by the fact that women…during lockdown": it is a strong sentence and despite sharable authors should provide some evidence to support;

Response: We have slightly rephrased this sentence to make it less direct. Importantly, we have now cited the work from Carli (2020) investigating the associations between gender inequality during the COVID-19 pandemic.

Carli, L. L. (2020). Women, gender equality and COVID-19. *Gender in Management: An International Journal, 35*(7/8), 647-655. https://doi.org/10.1108/GM-07-2020-0236

- In method and measures "Questionnaire can be found in supplementary materials" is repeated;

Response: Thank you for highlighting this redundancy. We have removed one of them.

- In results, the part: “To test this, we used the chi-square goodness-of-fit test including its adjusted standardized residuals to determine whether the multi-trajectory membership probabilities were higher for women (vs. men) and higher for younger adults (v. older adults). Specifically, cross-tabulations between four latent classes of compassionate self-responding trajectories and sociodemographic characteristics (i.e., age and gender) were conducted.” And “To reach our third goal, we used five univariate ANOVA to analyze the associations between multi-trajectory groups and negative emotions, happiness, sleep quantity and quality, and life satisfaction.” Is method, move to statistical modelling in method;

Response: We decided to change our analysis strategy for this analysis. We now rely on the MULTRISK function from PROC TRAJ in SAS software. This approach is more robust to capture longitudinal data and we successfully replicated our results with these new advanced techniques. Having said that, we have moved the methodological aspect of the analysis strategy in the method section to make it clear for the reader. Thank you for pointing this out.

- In discussion: “The general research question of the present paper was to investigate positive key factors that help people better cope with the psychological challenges associated with the COVID-19 pandemic.” Is not corresponding to the three objectives and hypothesis.

Response: Thank you for pointing out this imprecision. We have modified the sentence accordingly: “The main goal of the present paper was to investigate the positive facets of self-compassion as key factors that could help people better cope with the psychological challenges in the context of the COVID-19 pandemic.”

We then described in the same paragraph the three objectives and hypotheses.

- In discussion several sentences are too strong, such as “…compassionate self-responding were associated with greater life satisfaction…”: I suggest to use conditional using for example “more likely to be associated…);

Response: We were very careful not to include any causal language in the manuscript and have modified some sentences as you suggested to reflect the fact that observed associations may not replicate in another sample.

- In introduction authors cite “preventive intervention”, but none of them or future prospective are proposed in discussion section;

Response: Our results provided preliminary evidence supporting that self-compassion-based trainings can help people in difficult times. We developed this approach in the discussion section as an efficient intervention strategy. Following your suggestion, we have included a sentence in the subsection “Practice” in the “Implications and Future Directions” of the discussion section: “The current study contributes to practice by identifying self-compassion as a potential key factor associated with mental health that practitioners could exploit to develop and deliver preventive actions and effective interventions aimed at supporting people in times of crises (MacDonald & Neville, 2023).”

MacDonald, H. Z., & Neville, T. (2023). Promoting college students’ mindfulness, mental health, and self-compassion in the time of COVID-19: feasibility and efficacy of an online, interactive mindfulness-based stress reduction randomized trial. *Journal of College Student Psychotherapy, 37*(3), 260-278. https://doi.org/10.1080/87568225.2022.2028329

- In discussion the phrase “the greater variation observed for some individuals suggests that compassionate self-responding can also manifest as a state influenced by the global context as well as daily routines and life events.” Is too strong;

Response: As you suggested, we tempered this sentence: “On the other hand, the greater variation observed for some individuals suggested that compassionate self-responding may, to some extent, have also manifested as a state, influenced by the global context as well as daily routines and life events.”

- The citation Limcaoco refers to a paper in medRxiv in 2020. I think a paper not published in a peer-review journal should not be cited, especially if two-three years has passed;

Response: Thank you for pointing out this incorrect reference. We have included the published one and adjusted the text accordingly: "In a cross-national study involving 48 countries, Gamonal-Limcaoco et al. (2022) found high levels of negative emotions in the general population, with higher levels among women, young adults and those who expressed concern about getting infected."

Gamonal-Limcaoco, S., Montero-Mateos, E., Lozano-López, M. T., Maciá-Casas, A., Matías-Fernández, J., & Roncero, C. (2022). Perceived stress in different countries at the beginning of the coronavirus pandemic. *The International Journal of Psychiatry in Medicine, 57*(4), 309-322. https://doi.org/10.1177/00912174211033710

- Considering methods, authors stated that self-compassion scale is designed with 6 items, whereas in this study 3 items were used: I asking if the validity is guaranteed and I am not so confident. I appreciated the fact that authors discussed this particular point, justifying it with the interviewer engagement, but I think this aspect should be highlighted in method and discussion.

Response: We agree with your comment and believe this is a good point to consider. Following your suggestion, we have collected additional data to test for their convergent validity (see new Table 2). Specifically, we wrote: "To assess the convergent validity of the three positive facets of self-compassion, we conducted an additional data collection (Wave 11, $N = 1671$). This data collection included the three adapted single items tailored to the COVID-19 context, as well as their respective single items from the original scale. The correlation results presented in Table 2 demonstrated significant and strong associations between the COVID-19 items and the original items, supporting the convergent validity of our scale ($r_{\text{self-kindness}} = .56$, $p < .001$, $r_{\text{common humanity}} = .58$, $p < .001$, $r_{\text{mindfulness}} = .53$, $p < .001$)."

Although this comments, it is evident that authors did a lot of work in the writing and proofreading of the manuscript. Thank you again for allowing me to review your paper. I hope you will consider these revisions, as I think this paper does provide some really valuable information addressing an unmet need to understand the wellbeing of population and variables that can act on negative effects of COVID-19 pandemic on mental health.

Response: Thank you again for your very useful and constructive review. Based on your comments, we believe that our manuscript has substantially improved. We are looking forward to receiving your feedback on this newly improved version.

7. PLOS authors have the option to publish the peer review history of their article (what does this mean?). If published, this will include your full peer review and any attached files.

If you choose “no”, your identity will remain anonymous but your review may still be made public.

Do you want your identity to be public for this peer review? For information about this choice, including consent withdrawal, please see our Privacy Policy.

Reviewer #3: No

Reviewer #4: No

Reviewer #5: No

Reviewer #6: No

Reviewer #7: No

[NOTE: If reviewer comments were submitted as an attachment file, they will be attached to this email and accessible via the submission site. Please log into your account, locate the manuscript record, and check for the action link "View Attachments". If this link does not appear, there are no attachment files.]

While revising your submission, please upload your figure files to the Preflight Analysis and Conversion Engine (PACE) digital diagnostic tool, https://pacev2.apexcovantage.com/. PACE helps ensure that figures meet PLOS requirements. To use PACE, you must first register as a user. Registration is free. Then, login and navigate to the UPLOAD tab, where you will find detailed instructions on how to use the tool. If you encounter any issues or have any questions when using PACE, please email PLOS at figures@plos.org. Please note that Supporting Information files do not need this step.
