# Supplementary material for: Trajectories of self-kindness, common humanity, and mindfulness during the COVID-19 pandemic: A person-oriented multi-trajectory approach
Source: PLoS One. 2023 Dec 18;18(12):e0292522. doi: 10.1371/journal.pone.0292522 (PMC10727429; doi:10.1371/journal.pone.0292522)
Supplement: Attachment — Submitted filename: Peer_review_Manuscript Number PONE-D-22-01485.docx [file pone.0292522.s001.docx]

**Overall comments**

An interesting study with some good findings, just potentially not framed in the best way. In general, the paper could be re-written more concisely, with more targeted references and justification. There are some methodological concerns on how self-compassion has been operationalised, with limited evidence that the adapted measure has been tested for validity. I do feel that some more effort is needed to highlight the relevance of the work, how it will add to previous literature, and more work is needed to clearly present the results. The paper is framed as looking at the relationship between self-compassion and mental health over the COVID-19 pandemic, however, only positive elements of self-compassion, and outcomes *associated* with mental health are measured. Based on this I feel like the justification for the study set out in the introduction is not coherent with the study conducted. In the results, it is not clear how the trajectories of self-compassion changed over the pandemic. This could be presented more clearly so a person with minimal knowledge of trajectory modelling could understand where there were fluctuations in self-compassion over time, in which groups, and by how much. Results do indicate that those with high self-compassion also had better outcomes in factors associated with mental health, however, mental health was only recorded once so cause and effect is difficult to ascertain, which is a shame due to the longitudinal design.

More specific comments are written below.

**Introduction**

In general, the introduction is very long and needs to be made more concise. There is limited justification for why only compassionate responding is used and why factors associated with mental health outcomes are looked at rather than direct measures of mental health etc.

1) Neff (2003) is referenced for the conceptualisation of self-compassion and justification for the research, but the method does not measure self-compassion. It only measures levels of compassionate responding and ignores levels of uncompassionate responding. However, according to Neff, self-compassion consists of 3 sets of opposing elements to form a global self-compassion score (self-kindness vs. self-judgement, mindfulness vs. over identification and common humanity vs isolation). The introduction discusses the opposing elements of compassionate responding (e.g. self-judgement, isolation and over-identification) to form the justification for this research, yet these elements are not measured. There needs to be some justification in the introduction why this study only looks at the three positive elements of self-compassion.

2) The language used to describe self-compassion also needs changing to reference it as compassionate self-responding rather than self-compassion, as it currently implies that global self-compassion is being measured, e.g. :

“In addition, while it has been demonstrated that the combination of self-kindness, common humanity, and mindfulness reflects a global score of self-compassion (Neff et al., 2017)”

“…how each dimension of self-compassion changes over time…”

3) The majority of the research referenced in relation to mental health also uses the self-compassion scale which measures all 6 elements, not just 3. The evidence in relation to mental health needs to be more in line with the compassionate responding, rather than global self-compassion, to strengthen the argument.

4) The introduction is very focused on self-compassions relationship with mental health directly, including levels of depression, anxiety etc. But the study methods do not look at mental health directly, it looks at outcomes related to mental health (negative emotions, happiness, sleep quantity, sleep quality, and life satisfaction). The introduction should be tailored to discuss the link between self-compassion and those specific outcomes, rather than leaning so heavily on mental health specifically.

5) In general, the Self-Compassion and Sociodemographic Factors section would benefit from being reworded. It is quite confusing, and the justification for looking at age reads that younger people have worse mental health and therefore must have worse self-compassion, when there are lots of variables and factors that impact mental health in younger people differently. It would be better to take a softer approach and state that it is unknown whether self-compassion differs across age groups, which this study will explore.

6) “In the same vein,” is used repeatedly in the document. I suggest using different wording to improve readability.

7) I think the introduction would benefit from breaking the hypothesis and goals down into research questions. The goals of the study are clear, but it is unclear what questions will be answered if the goals are achieved.

**Method**

1) Demographic information would be better presented as a table. Age could be presented as a mean and range rather than percentage in age categories.
2) It would be good to include the year when the dates of the waves are given. The pandemic spanned multiple years so it is unclear whether the data was collected early in the pandemic.
3) Planned missingness – this section is quite passive. It is unclear how missingness was dealt with. It is mentioned that multiple versions of the questionnaire were used but it is unclear what this means? Do the questions change, the order, the wording?
4) The measures would benefit from some indices of validity and reliability.
5) I have some reservations about the self-compassion measures that were used.
   i. One aim of the study was to “examine the developmental trajectories of the three self-compassion dimensions during the COVID-19 crisis”. However, by adapting the wording of the measure what you are actually measuring is the level of compassionate self-responding in direct relation to suffering caused directly by COVID-19, not just general self-compassion levels “during” the COVID-19 crisis.
   ii. I raise this because the wording of the self-compassion items are difficult to interpret and I imagine participants interpretation of “related to the COVID-19 crisis” element of the items varied quite widely. Did you do any qualitative work with participants to explore their understanding of the question? If so, it would be good to include this to justify the wording.
   iii. Did you run any validity checks comparing responses to these items to items from the self-compassion scale?
   iv. Do you have a measure of reliability of these singular items?
   b. Usually standardised measures of mental health ask respondents about their mood or behaviour over a period of time. However, the measure of mood used here is a state-based measure rating current levels of particular emotions. This limits what conclusions can be drawn about mental health. For example, the person may have been really calm when filling in the questionnaire, but over the past two weeks had been highly worried.

**Results**

- Multinomial modeling of trajectories is not something I have read about before, so any comments come from the perspective of a lay reader.
  - I was expecting to see some analysis that demonstrated the degree of change in self-compassion over time from baseline, for each trajectory group.
  - It is mentioned that there are groups that show more persistent patterns, but there is no evidence that the change in pattern within the trajectory groups were significantly different between groups. It is unclear how the “more persistent pattern” was identified.
  - It is unclear from the results reported and the figures what the overall trajectories of self-compassion look like. It would be good to include a figure of each respondents overall self-compassion scores over time.
- The exploratory analysis does not seem necessary. It is not in the remit of the paper, and opens up a completely new goal, and set of interpretations that have not been discussed in the introduction. I would recommend removing this section.

**Discussion**

1) There are times that new information is built into the discussion that has not been mentioned in the rest of the paper. For example:
   a. *Strikingly, this peak corresponds to the shape of self-compassion trajectories, suggesting that this tremendous increased number of confirmed cases may have led to a decrease in self-kindness, common humanity and mindfulness over time (from wave 3 to wave 9), among individuals belonging to low trajectory groups –* this is not presented in the results section and is not displayed in any of the figures for the reader to confirm. Putting this in the results section in a figure to show the corresponding change in self-compassion would be beneficial.
   b. The discussion about self-compassion’s structure. This could be used in the intro as a justification for focusing on three components, but it does not fit well in the discussion.
2) Conclusions are drawn which suggest self-compassion is a stable trait and also a state, however from my interpretation of the results, there is limited evidence that self-compassion changes drastically. It seems from Figure 2 that respondents levels of self-compassion stay in the remit of their trajectory group. Therefore, this suggests that perhaps self-compassion levels may change slightly over time and circumstance, but that in general, the categorical level of self-compassion will stay the same, therefore adding more support for self-compassion as a trait then a state.
3) The paper is also limited by common method bias which can inflate correlation scores.
4) I would also suggest that paper is limited by measures of mental health outcomes only being measured at one time point. There is evidence that mental health outcomes fluctuated as self-compassion levels fluctuated.

**Other**

a. Figure 1 needs more detail to inform us about the data analysis plan. It does not inform us about IV/DV, excluded data, missing data, etc…
