# Supplementary material for: Trajectories of self-kindness, common humanity, and mindfulness during the COVID-19 pandemic: A person-oriented multi-trajectory approach
Source: PLoS One. 2023 Dec 18;18(12):e0292522. doi: 10.1371/journal.pone.0292522 (PMC10727429; doi:10.1371/journal.pone.0292522)
Supplement: Attachment — Submitted filename: Response to Reviewers.docx [file pone.0292522.s002.docx]

Reviewer #1:

Overall comments

An interesting study with some good findings, just potentially not framed in the best way. In general, the paper could be re-written more concisely, with more targeted references and justification. There are some methodological concerns on how self-compassion has been operationalised, with limited evidence that the adapted measure has been tested for validity. I do feel that some more effort is needed to highlight the relevance of the work, how it will add to previous literature, and more work is needed to clearly present the results. The paper is framed as looking at the relationship between self-compassion and mental health over the COVID-19 pandemic, however, only positive elements of self-compassion, and outcomes *associated* with mental health are measured. Based on this I feel like the justification for the study set out in the introduction is not coherent with the study conducted. In the results, it is not clear how the trajectories of self-compassion changed over the pandemic. This could be presented more clearly so a person with minimal knowledge of trajectory modelling could understand where there were fluctuations in self-compassion over time, in which groups, and by how much. Results do indicate that those with high self-compassion also had better outcomes in factors associated with mental health, however, mental health was only recorded once so cause and effect is difficult to ascertain, which is a shame due to the longitudinal design. More specific comments are written below.

**Response: We would like to thank Reviewer #1 for his/her detailed and useful comments. We believe the Reviewer raised excellent points that we aimed to address in this newly improved version. We also uploaded our comments, including updated figures, in a Word document to make it clearer for the reviewers. We anticipate your approval.**

Introduction

In general, the introduction is very long and needs to be made more concise. There is limited justification for why only compassionate responding is used and why factors associated with mental health outcomes are looked at rather than direct measures of mental health etc.

**Response: We have added a rationale for focusing on compassionate self-responding during the COVID-19 pandemic and for our choice of mental health indicators. Additionally, we have shortened the introduction, when possible.**

1) Neff (2003) is referenced for the conceptualisation of self-compassion and justification for the research, but the method does not measure self-compassion. It only measures levels of compassionate responding and ignores levels of uncompassionate responding. However, according to Neff, self-compassion consists of 3 sets of opposing elements to form a global self-compassion score (self-kindness vs. self-judgement, mindfulness vs. over identification and common humanity vs isolation). The introduction discusses the opposing elements of compassionate responding (e.g. self-judgement, isolation and over-identification) to form the justification for this research, yet these elements are not measured. There needs to be some justification in the introduction why this study only looks at the three positive elements of self-compassion.

**Response: In this study, we focused on the positive elements of self-compassion to untangle the role of compassionate self-responding from that of its negative aspects. We had a particular interest in the positive aspects of self-compassion because these aspects tend to be the focus of interventions to increase self-compassion through kindness, acceptance, and affection toward the self. This has been clarified throughout the paper.**

2) The language used to describe self-compassion also needs changing to reference it as compassionate self-responding rather than self-compassion, as it currently implies that global self-compassion is being measured, e.g. :

“In addition, while it has been demonstrated that the combination of self-kindness, common humanity, and mindfulness reflects a global score of self-compassion (Neff et al., 2017)”

“…how each dimension of self-compassion changes over time…”

**Response: As suggested, we have changed “self-compassion” to “compassionate self-responding” when necessary. For instance, the new title of the study is: “The dynamics of compassionate self-responding in Canadian adults during the first 8 months of the COVID-19 pandemic: A person-oriented multi-trajectory approach”. We adjusted the manuscript accordingly.**

**For example, we wrote in the introduction: “The purpose of the present research is threefold: (1) to identify trajectories of compassionate self-responding (i.e., self-kindness, common humanity, and mindfulness) during the first eight months of the COVID-19 pandemic based on 10 waves of data collection (from April 2020 to November 2020), (2) to examine associations between compassionate self-responding trajectories and sociodemographic variables (i.e., age and gender), and (3) to compare compassionate self-responding trajectories on key mental health indicators assessed at the end of this study, in a large representative sample of Canadians (*N* = 2458 from an initial sample size of 3617 participants). To the best of our knowledge, the current research is the first to investigate trajectories of compassionate self-responding in the context of dramatic social change (see de la Sablonnière, 2017).”**

3) The majority of the research referenced in relation to mental health also uses the self-compassion scale which measures all 6 elements, not just 3. The evidence in relation to mental health needs to be more in line with the compassionate responding, rather than global self-compassion, to strengthen the argument.

**Response: We now specify that past research has focused on both positive and negative elements of self-compassion.**

4) The introduction is very focused on self-compassions relationship with mental health directly, including levels of depression, anxiety etc. But the study methods do not look at mental health directly, it looks at outcomes related to mental health (negative emotions, happiness, sleep quantity, sleep quality, and life satisfaction). The introduction should be tailored to discuss the link between self-compassion and those specific outcomes, rather than leaning so heavily on mental health specifically.

**Response: We clarified that we focused on different mental health indicators and that these were chosen based on past research on self-compassion. More specifically, we now refer to outcomes related to mental health or indicators of mental health, rather than mental health directly.**

5) In general, the Self-Compassion and Sociodemographic Factors section would benefit from being reworded. It is quite confusing, and the justification for looking at age reads that younger people have worse mental health and therefore must have worse self-compassion, when there are lots of variables and factors that impact mental health in younger people differently. It would be better to take a softer approach and state that it is unknown whether self-compassion differs across age groups, which this study will explore.

**Response: The Self-Compassion and Sociodemographic Factors section has been revised according to the reviewer’s suggestion. We have decided to keep our hypotheses regarding the association with age and gender, but as suggested, we toned-down our claim significantly.**

6) “In the same vein,” is used repeatedly in the document. I suggest using different wording to improve readability.

**Response: “In the same vein” was written 4 times in the manuscript. We have modified this terms twice with “Similarly” and “In this context” to improve readability.**

7) I think the introduction would benefit from breaking the hypothesis and goals down into research questions. The goals of the study are clear, but it is unclear what questions will be answered if the goals are achieved.

**Response: As suggested, in the section « Overview of the Present Study and Hypothesis, we have clarified the general purpose of the study. For each hypothesis, we provided a clear research question that aims to be answered.**

Method

1) Demographic information would be better presented as a table. Age could be presented as a mean and range rather than percentage in age categories.

**Response: Because we are using age categories in our analysis, we have decided to present this variable in two formats: means and percentages. Means are reported in Table 1 and percentages are indicated within the method section. We followed a similar presentation for the gender variable.**

2) It would be good to include the year when the dates of the waves are given. The pandemic spanned multiple years so it is unclear whether the data was collected early in the pandemic.

**Response: As suggested, we have included the year when the dates of the waves are given both in the text and in Table 1.**

3) Planned missingness – this section is quite passive. It is unclear how missingness was dealt with. It is mentioned that multiple versions of the questionnaire were used but it is unclear what this means? Do the questions change, the order, the wording?

**Response: We have completely rewritten this section which is now titled: “Planned Missingness and Missing Values”. Specifically, we wrote: “Longitudinal data collection is a time-consuming and cost-intensive part of developmental research. In order to improve the validity of data collection, we relied on multi-form designs of planned missingness (for an overview, see Wu & Jia, 2021). Specifically, planned missing data designs allow researchers to collect incomplete data from participants by randomly assigning participants to have missing items on a survey. Following best practice procedures, we used several different versions of the same questionnaire for which each participant completed two-thirds of the total number of items. Note that the variables and wording remained exactly the same. This multi-form design is most useful when one wants to collect data using a large number of variables, but is faced with time constraints or concerns about respondent burden and fatigue (Rhemtulla et al., 2016).**

**Additionally, in group-based trajectory modeling, missing data (including planned missingness) are handled via the Full Information Maximum Likelihood (FIML) method. This allows for the inclusion of participants who have missing data on the dependent variables (Jones et al., 2001; Nagin, 1999). In sum, by correctly implementing these multi-form designs and properly dealing with missing values using appropriate approaches (e.g., Maximum Likelihood), the cost of data collection is lowered and reduced participant burden may result in higher validity as well as lower rates of unplanned missing data.”**

4) The measures would benefit from some indices of validity and reliability.

**Response: For single items, we reported the correlations and for multiple items, we have now reported the Omega’s alphas, including for negative emotions, which we omitted in the initial version. Thank you for pointing out this omission. Additionally, we also reported the correlation between happiness and negative emotions. Overall, all these indices reinforced the validity of our measures.**

5) I have some reservations about the self-compassion measures that were used.
   i. One aim of the study was to “examine the developmental trajectories of the three self-compassion dimensions during the COVID-19 crisis”. However, by adapting the wording of the measure what you are actually measuring is the level of compassionate self-responding in direct relation to suffering caused directly by COVID-19, not just general self-compassion levels “during” the COVID-19 crisis.

**Response: The reviewer is right in saying that we studied participants’ self-compassionate responding toward events related to the COVID-19 crisis specifically. The entire paper (including the title and abstract) has been revised accordingly.**

ii. I raise this because the wording of the self-compassion items are difficult to interpret and I imagine participants interpretation of “related to the COVID-19 crisis” element of the items varied quite widely. Did you do any qualitative work with participants to explore their understanding of the question? If so, it would be good to include this to justify the wording.

**Response: We agree that the manuscript did not clearly state that we assessed participants’ self-compassionate responding to events related to the COVID-19 crisis specifically, and not self-compassion in general. This has been clarified throughout the manuscript. We also added a rationale for focusing specifically on compassionate self-responding during the current COVID-19 pandemic. Please note that all the items used targeted this construct of compassionate self-responding “during the current COVID-19 pandemic”, regarding “their reaction to the COVID-19 pandemic” or “when something happens to them related to the COVID-19 crisis” specifically. The high correlations between the three adapted items suggested that participants also felt that these items assessed a clear common construct.**

iii. Did you run any validity checks comparing responses to these items to items from the self-compassion scale?

**Response: Given that the items evaluating self-kindness, common humanity, and mindfulness were framed in the context of the COVID-19 pandemic, we did not measure the original items. We included this as a limitation in the discussion. Importantly, we ran correlations between items and found that self-kindness was positively correlated with common humanity (*r* = .50, *p* < .001) and mindfulness (*r* = .48, *p* < .001) and the two latter were positively correlated with each other (*r* = .38, *p* < .001) at wave 1. Similar findings were observed for each of the other waves. As stated above, this suggests that these items are assessing the same variable.**

iv. Do you have a measure of reliability of these singular items?

**Response: Yes, we do. In fact, we added McDonald's omegas for each measure, including for the compassionate self-responding items. The internal consistency ranged from acceptable to good for the compassionate self-responding and were excellent for the dependent variables.**

b. Usually standardized measures of mental health ask respondents about their mood or behaviour over a period of time. However, the measure of mood used here is a state-based measure rating current levels of particular emotions. This limits what conclusions can be drawn about mental health. For example, the person may have been really calm when filling in the questionnaire, but over the past two weeks had been highly worried.

**Response: This is an important point to consider. It is pertinent to note that some of our indicators are capturing participants’ responses over a period of time. For instance, regarding sleep quantity, we** **asked participants how much sleep, in hours and minutes, did they get in the last 24 hours. Similarly, and for sleep quality, we measured the subjective qualitative facet of sleep by asking participants how they would describe the quality of their sleep during the last 24 hours. However, we agree with you that this could have been done for emotions as well. Consequently, we added a limitation in the discussion. Specifically, we wrote: “Moreover, standardized measures of mental health usually ask participants to provide their answers over a specific period of time. While we asked participants to describe the quantity and quality of their sleep within the last twenty-four hours, we only relied on a state-based measure rating current levels of particular emotions, which limits what conclusions can be drawn about mental health.”**

Results

- Multinomial modeling of trajectories is not something I have read about before, so any comments come from the perspective of a lay reader.
  - I was expecting to see some analysis that demonstrated the degree of change in self-compassion over time from baseline, for each trajectory group.

**Response: This is an interesting point to consider. The paper of van der Nest et al. (2020) offers a very detailed and clear approach to different longitudinal growth models. In the present paper, we relied on a person-oriented multi-trajectory approach which is a group-based trajectory method from a latent class grow analysis (see Figure 1 in van der Nest et al., 2020). Doing so, we were not focusing on a difference of change from the baseline, but rather on fluctuation of self-compassion items between waves 1 and 10. We believe this approach provided more information on the dynamics of each measured construct.**

**van der Nest, G., Passos, V. L., Candel, M. J., & van Breukelen, G. J. (2020). An overview of mixture modelling for latent evolutions in longitudinal data: Modelling approaches, fit statistics and software. *Advances in Life Course Research, 43*, 100323. https://doi.org/10.1016/j.alcr.2019.100323**

- It is mentioned that there are groups that show more persistent patterns, but there is no evidence that the change in pattern within the trajectory groups were significantly different between groups. It is unclear how the “more persistent pattern” was identified.
- It is unclear from the results reported and the figures what the overall trajectories of self-compassion look like. It would be good to include a figure of each respondents overall self-compassion scores over time.

**Response: Our search for “more persistent pattern” in the manuscript did not show any results. So, it is difficult to identify and address the Reviewer’s comment. Furthermore, it is pertinent to mention that we relied on multiple fit indices to identify statistically distinct trajectory groups (e.g., BIC, AIC). Additionally, we sometimes wrote that “This group […] was characterized by a negative linear function, suggesting that these low trajectories were more unstable and decreased over time”. In this context, we assume that a linear function is more fluctuating than a constant function. Ultimately, we do not assume that groups show “more persistent patterns”.**

**In addition, we have reworked Fig 2 to make it clearer for the reader. Please see below.**

**Self-Kindness**

**Common Humanity**

**Mindfulness**

**Fig 2.** **Multi-trajectory groups with 95% CI (dashed lines) by dimensions.** **Participants experiencing low (*N* = 97, 3.9% in purple), moderate-low (*N* = 965, 39.3% in orange), moderate-high (*N* = 1149, 46.7% in blue) and high (*N* = 247, 10.0% in green) levels across the three dimensions.**

- The exploratory analysis does not seem necessary. It is not in the remit of the paper, and opens up a completely new goal, and set of interpretations that have not been discussed in the introduction. I would recommend removing this section.

**Response: We agree with you that the exploratory analysis is not necessary and might distract the reader from the main goals of the paper. We have thus moved this analysis in the online supplementary material.**

Discussion

1) There are times that new information is built into the discussion that has not been mentioned in the rest of the paper. For example:
   a. *Strikingly, this peak corresponds to the shape of self-compassion trajectories, suggesting that this tremendous increased number of confirmed cases may have led to a decrease in self-kindness, common humanity and mindfulness over time (from wave 3 to wave 9), among individuals belonging to low trajectory groups –* this is not presented in the results section and is not displayed in any of the figures for the reader to confirm. Putting this in the results section in a figure to show the corresponding change in self-compassion would be beneficial.

**Response: As suggested, we have moved this figure in the results section and adjusted the text accordingly. We have also improved the figure to make it clearer for the reader. Specifically, we replaced the red dots with a grey zone. Ultimately, we placed this figure next to the trajectories to facilitate the comparison. We believe that merging these figures into a single graph would make it complicated for the reader.**

**Fig 3.** **Vaccine doses, daily new confirmed cases, ICU patients, and death cases in Canada. The grey zone indicates the period of our data collection.**

b. The discussion about self-compassion’s structure. This could be used in the intro as a justification for focusing on three components, but it does not fit well in the discussion.

**Response: Given the nature of our measure, we have significantly toned-down the debate surrounding the structural aspect of the self-compassion measure. However, please note that we have better justified the choice of the three positive components.**

2) Conclusions are drawn which suggest self-compassion is a stable trait and also a state, however from my interpretation of the results, there is limited evidence that self-compassion changes drastically. It seems from Figure 2 that respondents levels of self-compassion stay in the remit of their trajectory group. Therefore, this suggests that perhaps self-compassion levels may change slightly over time and circumstance, but that in general, the categorical level of self-compassion will stay the same, therefore adding more support for self-compassion as a trait then a state.

**Response: We agree with you that compassionate self-responding is rather stable over time and variations are minor. We revised the manuscript accordingly. For example, we wrote in the results section: “Taken together, these results indicated that compassionate self-responding dimensions were rather stable over time with minor fluctuations and diminutions for some individuals.”**

3) The paper is also limited by common method bias which can inflate correlation scores.

**Response: As suggested, we have included an additional limitation. Specifically, we wrote: “In addition, it is pertinent to note that the correlational design could have generated common method bias. These bias may occur in studies where data for both independent and dependent variables are obtained from the same person in the same measurement context, using the same item content, and similar item characteristics. In the present study, these bias might have inflated observed correlations.”**

4) I would also suggest that paper is limited by measures of mental health outcomes only being measured at one time point. There is evidence that mental health outcomes fluctuated as self-compassion levels fluctuated.

**Response: This is a good suggestion. We included this aspect in the limitation: “Similarly, the present research is limited by measures of mental health outcomes only being measured at one time point. There is evidence that mental health outcomes may fluctuate in relation to changes in self-compassion. Future research should investigate trajectories of mental health indicators and explore their conditional probabilities with self-compassion trajectories.”**

Other

a. Figure 1 needs more detail to inform us about the data analysis plan. It does not inform us about IV/DV, excluded data, missing data, etc…

**Response: We have improved Fig 1 based on the new modifications. To goal of this figure is to prepare the reader for the subsequent analysis. Independent and dependent variables are clearly indicated on the figure. All information about missing data are now available in Table 1 as well as in the new Planned Missingness and Missing Values section.**

**Fig 1. Data analysis plan.**

**Ultimately, we would like to thank very much Reviewer 1 for his/her valuable and helpful feedback to improve our manuscript. We sincerely anticipate that you will approve this new version.**

Reviewer #2:

The authors should be commended with respect to the clarity of their writing; it is clear that a lot of work has gone into the proofreading and editing of this document before submission, which has made the review process much easier. In terms of the technical processes taken, a lot of what the authors have conducted is on the right track, therefore I will just describe the parts of their methodology and conclusions that may benefit from a second pass. I had a few queries about the specifics of the analysis for the authors to consider, which may have an impact on their choice of model and the conclusions drawn.

**Response: We would like to thank Reviewer #2 for his/her very encouraging and constructive feedback. We are particularly grateful to this reviewer for his suggestions as he is an expert in longitudinal methods.**

1) The authors seem to rely on BIC and BIC alone when making decisions on the best-fitting model to the data ("Based on the BIC criterion, a four-group model was selected as the best fitting model."). Mixture models have an array of model adequacy criteria to peruse as the investigator, however. I would appreciate it if the authors not only presented their BIC-based conclusions, but also considered the array of additional model adequacy criteria regarding the count of classes. As outlined in excellent papers that address this issue specifically such as van der Nest et al. (2020; https://doi.org/10.1016/j.alcr.2019.100323), other coefficients such as the Vuong-Lo-Mendell-Rubin Likelihood Ratio Test are available to help inform decisions related to the count of classes that is optimal. Noting that the authors are using PROC TRAJ in SAS, and as noted in van der Nest et al. the fit coefficients are similar to Stata in being very limited when informing this part of model selection, I am curious if the authors can describe other aspects of class enumeration that they considered? For example, Nylund-Gibson and Choi's (2018; http://dx.doi.org/10.1037/tps0000176) general tutorial article on class enumeration in an LCA context describes considerations such as the minimum proportion of participants estimated to be members of a class in order to help with establishing class stability. They provide an example of asking whether a small proportion of the sample captured by a class is like to be stable, using a rough rule of thumb of approximately 5% as something large enough. Alternatively, Muthen's presentations on mixture models provide examples of valid latent classes with <5% of the sample as being valid. I think the authors need to flesh out their model selection considerations beyond that of just the BIC coefficient, keeping in mind the kinds of considerations discussed prior, to show some additional nuance in their decision making prior to interpretation. Employing the D’Unger et al. (1998) resource to inform model selection from over two decades ago isn't adequate given the evolution of mixture modelling in the meantime.

**Response: This is an excellent point to consider. We selected our main model based on two criteria. First, we indeed relied on the BIC from D’Unger et al. (1998). Second, we selected our model based on a power calculation. We have added this information: “It is pertinent to note that because models including five or six trajectory groups generated trajectories containing a limited number of participants, we selected the four-group model containing sufficient participants in each latent clusters to provide appropriate power to conduct subsequent analyses. Specifically, Jung and Wickrama (2008) argued that when running latent class growth analysis models, at least 1% of the total sample should comprise each class; however, this rule depends on the sample size. The smaller cluster of the four-group model contains 3.9% that translates into 97 participants which generates sufficient power to detect the significance of slopes within trajectory classes.”**

**We appreciate the recommendation from the Reviewer to include additional model fit statistics. To stay consistent with our statistical software program (PROC TRAJ in SAS), we have now included an additional and accessible indice, the Akaike information criterion (AIC) which confirmed the 4-group model (see Table 2).**

2) Can the authors cite the basis of their interpretation of the posterior assignment probabilities as being adequate when over >.70? The van der Nest article I've cited prior might help them do this, specifically the section on APPA. Currently the values are presented without justification.

**Response: This is an excellent recommendation. We have improved this section and wrote: “The average posterior probability of assignment (APPA; Asparouhov & Muthén, 2014) estimating the probability of belonging to each trajectory group conditional on longitudinal responses is reported in Table 3. The APPA is calculated as the average posterior probability of belonging to class k over all the individuals assigned to class k (van der Nest et al., 2020). When posterior probabilities are near 100% there is minimal classification error because participants assigned to a specific trajectory have a maximal chance of belonging to that trajectory. Ideally, this probability should be near 100% for the assigned group and, conversely, near 0% for the rest, with a minimum threshold of 70% (Nagin, 2005). The average probabilities for the assigned groups are respectively 92%, 89%, 90% and 94%, indicating low classification error.”**

3) When looking at the associations between the estimated (and I would emphasise the latter term) class assignments and sociodemographic covariates / distal outcomes, the authors seem to be employing a one-step approach. Much of the recent literature on covariate / distal outcome prediction involving mixture models has heavily cautioned against employing this approach. The authors should refer to the summary provided by Asparouhov and Muthén (2014; https://doi.org/10.1080/10705511.2014.915181) for a description of the shortcomings of the one-step approach, which have been addressed by the contemporary three-step approaches to covariate and distal outcome estimation. If the three-step BCH approach for distal outcome estimation (i.e., for the mental health outcomes based on latent class), or the three-step covariate estimation approach (i.e., for sociodemographic variables) are not available in SAS, then the authors will need to concretely defend the validity of their proposed associations between the latent classes and covariates/outcomes.

**Response: With respect to Reviewer #2, it is important to note that we relied on a two-step method to assess the role of each sociodemographic and outcome variables. Specifically, step 1 identified the multi-trajectory group, whereas steps 2 and 3 involved exporting the classification and conducting subsequent analyses independently from the latent class grow analysis used in step 1.**

4) The final sentence of the Results is a bit of a mystery - what did you find? Was it the same when MI was employed? How many imputations did you run, etc.?

**Response: We agree with you that the last sentence of the results section was vague. We initially simplified this part to stay within the word limit. We also believe that these multiple imputation techniques served as a successful control check, but might distract the reader from the main objectives. Consequently, we have made it clearer by creating a new “Planned Missingness and Missing Values” part in the method section: “Longitudinal data collection is a time-consuming and cost-intensive part of developmental research. In order to improve the validity of data collection, we relied on multi-form designs of planned missingness (for an overview, see Wu & Jia, 2021). Specifically, planned missing data designs allow researchers to collect incomplete data from participants by randomly assigning participants to have missing items on a survey. Following best practice procedures, we used several different versions of the same questionnaire for which each participant completed two-thirds of the total number of items. Note that the variables and wording remained exactly the same. This multi-form design is most useful when one wants to collect data using a large number of variables, but is faced with time constraints or concerns about respondent burden and fatigue (Rhemtulla et al., 2016).**

**Additionally, in group-based trajectory modeling, missing data (including planned missingness) are handled via the Full Information Maximum Likelihood (FIML) method. This allows for the inclusion of participants who have missing data on the dependent variables (Jones et al., 2001; Nagin, 1999). In sum, by correctly implementing these multi-form designs and properly dealing with missing values using appropriate approaches (e.g., Maximum Likelihood), the cost of data collection is lowered and reduced participant burden may result in higher validity as well as lower rates of unplanned missing data.”**

5) Referring to non-linear trajectories as an 'unstable shape' is tricky within the context of mixture models. This may be interpreted as the scaled entropy of the trajectory class becoming poorer over time, for example. Consider referring to these trajectories as non-linear.

**Response: This is a great recommendation. We agree with you that unstable shape is vague and can be misleading. As suggested, we have replaced “unstable” with “non-linear” when necessary.**

6) When viewing the plots of the trajectories, their non-linearity seems minor. Furthermore, the generally flat trajectories of the classes makes me wonder how the LCGA models compare with the much simpler growth models that don't split the trajectories within the sample. Acknowledging that the red class (3.2% of the sample) appears to be the lone class that isn't flat in trajectory shape, and is relatively marginal in the count of participants classified in this class, can the authors compare their findings with a generic growth model that will likely turn out to be relatively flat across the waves of measurement? There's a parsimony consideration here, and while person-centered analyses provide the opportunity for additional detail about these trajectories, the value of these distinct classes versus being able to describe people scoring high/med-high etc. over time based on a lone flat trajectory is worth considering in the Discussion.

**Response: One goal of our paper was to discover the presence of heterogeneity of self-compassion through time during the COVID-19 pandemic. The use of a growth curve model would not have satisfied that goal. Also, the fact that the identified trajectories are mostly linear does not imply that a growth curve model would have been a better type of analysis. On the contrary, our results support the presence of heterogeneity in the self-compassion trajectories as four trajectories were identified. Furthermore, our aim was not to determine the most parsimonious model to study self-compassion. Overall, we do not think that estimating a growth curve model would be beneficial to our goal and to our paper. However, and in line with your comment, we have revised the discussion regarding the overall minor non-linearity of our trajectories. In addition, we have reworked all figures to make them clearer for the readers.**

I hope these comments/queries have been useful in helping with your publication, and thank you for the opportunity to provide reviewer comments.

**Response: We would like to thank you again for your wisdom and guidance. We wish our newly improved version will meet your expectations.**
