# Supplementary material for: Trajectories of self-kindness, common humanity, and mindfulness during the COVID-19 pandemic: A person-oriented multi-trajectory approach
Source: PLoS One. 2023 Dec 18;18(12):e0292522. doi: 10.1371/journal.pone.0292522 (PMC10727429; doi:10.1371/journal.pone.0292522)
Supplement: Attachment — Submitted filename: Manuscript Number PONE-D-22-01485.odt [file pone.0292522.s003.odt]

I don’t believe any of my initial review points have been taken on board or edited. I have included examples of this below. As these points have not been addressed, either through edits in the document or through responses from the authors with defence as to why these decisions were made I want to recommend that this paper is not published. I do not trust that these authors have a full grasp of what self-compassion is and have not put thought behind the operationalisation of the concepts of self-compassion or mental health and therefore cannot justify why they have used them. If a lay person was to read this paper, they would learning inaccurate information about what self-compassion is and how it is defined. There has been no effort to rectify this from when I initially reviewed the paper. 
The above is particularly important because the authors have edited the self-compassion measure and have only measured the three elements. As the self-compassion scale is designed to measure all 6 facets with specific wording, I am unconfident on the validity of this measure but some justification in the introduction may have helped alongside some pilot analysis showing that the measure is associated with outcomes from the SCS - but I am still very sceptical about the validity and reliability of this measure

In turn I have concerns about have conclusions drawn on how self-compassion protected mental health during the pandemic. Self-compassion was measured across 10 waves, but we have no clear indication of attrition here, and mental health was recorded at one time point. An improved study would have measured mental health at each time point and looked to understand how mental health fluctuated with self-compassion. 

Point 1: Introduction
Comments from my first review:
“1) Neff (2003) is referenced for the conceptualisation of self-compassion and justification for the research, but the method does not measure self-compassion. It only measures levels of compassionate responding and ignores levels of uncompassionate responding. However, according to Neff, self-compassion consists of 3 sets of opposing elements to form a global self-compassion score (self-kindness vs. self-judgement, mindfulness vs. over identification and common humanity vs isolation). The introduction discusses the opposing elements of compassionate responding (e.g. self-judgement, isolation and over-identification) to form the justification for this research, yet these elements are not measured. There needs to be some justification in the introduction why this study only looks at the three positive elements of self-compassion.” 

2) The language used to describe self-compassion also needs changing to reference it as compassionate self-responding rather than self-compassion, as it currently implies that global self-compassion is being measured, e.g. :

“In addition, while it has been demonstrated that the combination of self-kindness, common humanity, and mindfulness reflects a global score of self-compassion (Neff et al., 2017)”

These points just have not been addressed. The same text is included in the introduction and no effort has been applied to justify their choices for only focusing on the positive elements of self-compassion.  As such, I have grave concerns about the theoretical and conceptual understanding of the work. The definition provided for self-compassion is inaccurate, stating that there are only 3 facets of self-compassion, and not six as clearly conceptualised in Neff 2003b. This reference has been used to support the definition provided in this paper and it is inaccurate. If the paper wanted to focus on the positive facets of self-compassion, this would be fine and Neff (2018) states that people can focus on either compassionate responding or uncompassionate responding independently if this fits the aim of their work, however this paper has not justified why they are only focusing on positive elements and have not considered the implications of this at any point in their paper. This is poor scientific practice. If people were to read this article alone without understanding of self-compassion, their teachings would be inaccurate and biased to the author’s cause. As this comment hasn’t been addressed, and the definition of self-compassion has just been changed to remove the negative elements this suggests the authors have no justification for not including the negative elements which makes me concerned about their understanding and interpretation of the findings. 
Only including the positive elements of self-compassion makes the data vulnerable to common method bias and inflated ITT.  
Methodology, Results and Discussion
It is still unclear how low, moderate (high & low) and high self-compassion were determined. What were the cut off points? How were these decided. This still hasn’t been addressed from my first review. As the measure is not in line with the validated measure of self-compassion I would like to fully understand how cut offs were determined and how this linked to the cut offs associated with the validated self-compassion scale. 

There is no discussion of how assumptions were checked. 

As there was a high attrition rate, how was this dealt with in the analysis? 

Other comments not addressed from first review:
I would have expected these points to be added to the limitations section:
3) The paper is also limited by common method bias which can inflate correlation scores – particularly as only positive elements of the SCS were used.
4) I would also suggest that paper is limited by measures of mental health outcomes only being measured at one time point. There is evidence that mental health outcomes fluctuated as self-compassion levels fluctuated.
